# Quantitative trait loci underlying resistance to sudden death syndrome (SDS) in MD96-5722 by ‘Spencer’ recombinant inbred line population of soybean

**DOI:** 10.1007/s13205-014-0211-3

**Published:** 2014-04-30

**Authors:** J. Anderson, M. Akond, M. A. Kassem, K. Meksem, S. K. Kantartzi

**Affiliations:** 1 Department of Plant, Soil and Agricultural Systems, Southern Illinois University, Carbondale, IL 62901-4415 USA; 2 Plant Genomics and Biotechnology Lab, Department of Biological Sciences, Fayetteville State University, Fayetteville, NC 28301-4298 USA

**Keywords:** Sudden death syndrome, Inbred lines, Quantitative trait loci

## Abstract

The best way to protect yield loss of soybean [*Glycine max* (L.) Merr.] due to sudden death syndrome (SDS), caused by *Fusarium virguliforme* (Aoki, O’Donnel, Homma & Lattanzi), is the development and use of resistant lines. Mapping quantitative trait loci (QTL) linked to SDS help developing resistant soybean germplasm through molecular marker-assisted selection strategy. QTL for SDS presented herein are from a high-density SNP-based genetic linkage map of MD 96-5722 (a.k.a ‘Monocacy’) by ‘Spencer’ recombinant inbred line using SoySNP6K Illumina Infinium BeadChip genotyping array. Ninety-four *F*_5:7_ lines were evaluated for 2 years (2010 and 2011) at two locations (Carbondale and Valmeyer) in southern Illinois, USA to identify QTL controlling SDS resistance using disease index (DX). Composite interval mapping identified 19 SDS controlling QTL which were mapped on 11 separate linkage group (LG) or chromosomes (Chr) out of 20 LG or Chr of soybean genome. Many of these significant QTL identified in one environment/year were confirmed in another year or environment, which suggests a common genetic effects and modes of the pathogen. These new QTL are useful sources for SDS resistance studies in soybean breeding, complementing previously reported loci.

## Introduction

Sudden death syndrome (SDS) caused by *Fusarium virguliforme* (Aoki, O’Donnel, Homma & Lattanzi) (Aoki et al. 2003) is one of the most devastating diseases of soybean (*Glycine max* L. Merr.) responsible for severe yield loss. SDS spreads quickly across the Americas and became a major pest problem for soybean growers starting in the 1980s (Wrather et al. 2003; Roy 1997), but it was not known in Asia until 2011 (Srour et al. 2012). Yield loss ranges between 5 and 15 %, while trace losses can go up to 80 %, depending on environmental conditions, genotype, and planting date (Ohmes 2013). SDS genetics are complex because it is multi-genetic (Anderson 2012; Kazi et al. 2008) and the environmental-controlled nature of disease (Gongora-Canul 2010). *F. virguliforme* is capable of causing root rot that affects root mass and produces a toxin that is responsible for foliar symptoms (Njiti et al. 1998; Kazi et al. 2008). Some of soybean cultivars have a dual resistance to SDS leaf scorch and root infection (Njiti et al. 1997, 2001, 2003; Hartman et al. 1997) such as ‘Forrest’ (Hartwig and Epps 1973) and ‘Ripley’ (Njiti et al. 1997), but more genotypes need to be explored as sources of resistance (Hartman et al. 1997; Rupe et al. 1989; Iqbal et al. 2001). Although, the development of resistant varieties with high-yield performance requires considerable effort and time, it is considered the best approach to manage SDS (Westphal et al. 2008).

Genomic-assisted breeding using molecular techniques and quantitative trait loci (QTL) for cultivar screening shows promise. Previous reported projects screened or developed genotypes with resistance to SDS and identified important QTL closely linked to resistance genes. Stephens et al. (1993) identified a single dominant gene (*Rfs*) in soybean variety ‘Ripley’, while another locus for resistance to root infection (*Rfs1*) was detected on linkage group (LG) G in ‘Essex’ by ‘Forrest’ population. In the same population, Hnetkovsky et al. (1996), Chang et al. (1996) and Kassem et al. (2006) reported QTL conferring SDS on nine LG, e.g., A2, C2, D2, F, G, I, J, L and N. There are other QTL found on LG-A2 in ‘Ripley’ by ‘Spencer’ (Farias-Neto et al. 2006), on LG-L in ‘Minsoy’ × ‘Noir 1’ and on LG-H in ‘Essex’ by ‘Forrest’ populations of soybeans. However, currently SoyBase (2010): the Soybean Breeder’s Toolbox includes more than 56 detections of QTL for SDS, which identified in different populations. Identification of more QTL and DNA markers linked to SDS is worthwhile for cultivar development via marker-assisted selection (MAS).

Although simple sequence repeat (SSR) markers were used successfully for localizing QTL in soybeans (Hnetkovsky et al. 1995; Kassem et al. 2006; Kazi et al. 2008), single nucleotide polymorphism (SNP) markers are the most ample genetic markers available for taking research to the next level (Nicod and Largiader 2003; Fan et al. 2003; Barbazuk et al. 2007; Hsu et al. 2008). Currently, very few SNP markers/QTL are available for SDS resistance in soybean. Recently, Kassem et al. (2012) mapped 14 significant QTL linked to SDS resistance through a high-density SNP-based genetic linkage map using PI438489B by ‘Hamilton’ RIL population of soybean. Another SNP-based high-density genetic linkage map (Akond et al. 2013) was constructed using MD 96-5722 × ‘Spencer’ RIL population to map QTL for SDS resistance. Therefore, the objective of this research was to identify QTL conferring SDS resistance in a population advanced from a cross between soybean line MD 96-5722 and variety ‘Spencer’.

## Materials and methods

### Plant material

Ninety-four *F*_5:7_ lines generated by crossing a resistant line, MD 96-5722 (a.k.a Monocacy), with a susceptible line, Spencer (Wilcox et al. 1989), were evaluated against SDS by growing the population at Carbondale (2010) and Valmeyer (2010 and 2011) in southern Illinois, USA. Detailed information of parental lines and methods of development of RIL population can be found in Akond et al. (2013).

### Screening of SDS symptoms and data analysis

SDS leaf symptoms were rated and compared to two checks, one resistant, ‘Ripley’ (Cooper et al. 1990), and one susceptible, ‘Spencer’ (Wilcox et al. 1989), as close as possible to the R_6_ stage (Fehr et al. 1971) when seeds fill the pod cavity, but are not yet entered to senescence. SDS was rated by two scores; disease incidence (DI), which is the percentage of plants with SDS symptoms in a plot, and disease severity (DS). DS is rated on a 1–9 scale with 1 describing mild symptoms and 9 being the premature death of the plant: (1) 0–10 % where 1–5 % of leaf surface chlorotic/necrotic, (2) 10–20 % where 6–10 % of leaf surface chlorotic/necrotic, (3) 20–40 % where 10–20 % of leaf surface chlorotic/necrotic, (4) 40–60 % where 20–40 % of leaf surface chlorotic/necrotic, (5) >60 % where more than 40 % of leaf surface chlorotic/necrotic, (6) up to 33 % premature defoliation, (7) up to 66 % premature defoliation, (8) >66 % premature defoliation, and (9) premature death of plant. These two scores were used to calculate disease index (DX) with the formula (DI × DS)/9 (Njiti et al. 1996) and was used for QTL analysis. Heritability estimates and variation of SDS were calculated using the linear–bilinear model, i.e., additive main effects and multiplicative interactions (AMMI) function of JMP Genomics 6 (SAS Institute, Cary, NC). Main effects that tested were environment, replication, genotype, and their interactions.

### Genetic map and QTL identification

MD 96-5722 by ‘Spencer’ RIL population was genotyped using 5,376 SNPs through SoySNP6K Illumina Infinium BeadChip array. The genetic linkage map (Akond et al. 2013) was constructed through JoinMap 4 (Kyazma BV, Wageningen, Netherlands; Van Ooijen 2001). Composite interval mapping (CIM) was used to detect QTL from genotypic and phenotypic data using WinQTLCart 2.5 software (Wang et al. 2005). Model 6 with four parameters for forward and backward stepwise regression, 10 cM window size, 1 cM step size and five (5) control markers were chosen for running WinQTLCart (Wang et al. 2005). Threshold was determined by permutations in 1,000 times.

## Results

**Fig. 1:**
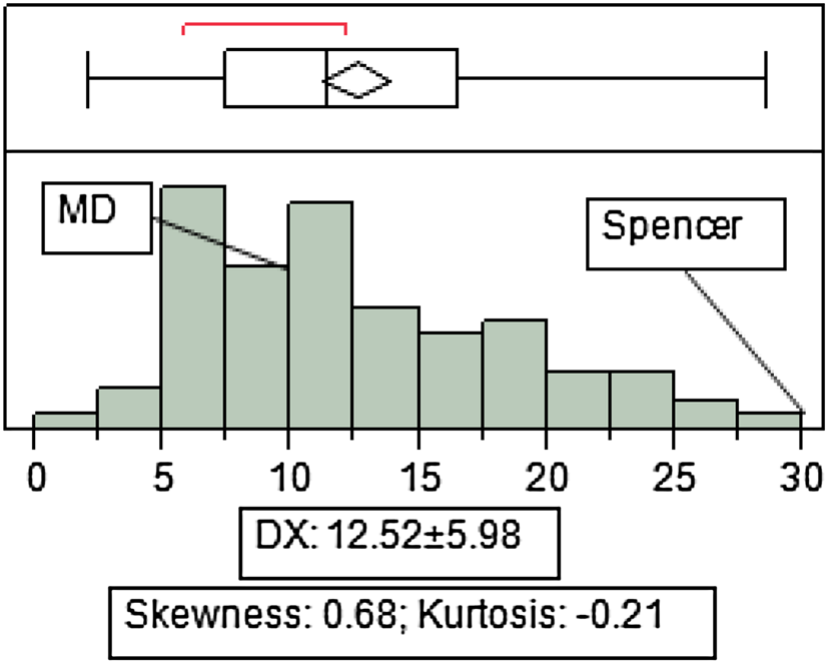
Phenotypic distribution of mean values of disease index (DX) from three different growing environments (Carbondale, IL in 2010 and Valmeyer, IL in 2010 and 2011) of MD 96-5722 and ‘Spencer’ recombinant inbred line (RIL) population. Mean values and standard deviation (mean ± SD) of DX, skewness and kurtosis are shown at the *lower* part of figure

Frequency distribution of DX was nearly normal as skewness and kurtosis values were <1.00 (Fig. 1). Mean squares of environment (E), replication (R), and genotype (G) were significant at *P* ≤ 0.0001, while G × E interaction was significant at *P* ≤ 0.005 for DX (Table 1). DX variation was 47.9 % and heritability was as low as 0.20, which is probably the consequence of high environmental variances (Table 2).

**Table 1:** G × E interaction pattern from the additive main effect and multiplicative interaction (AMMI) of RIL population of disease index (DX); RIL population was grown in three different environments i. e., Carbondale, IL in 2010, Valmeyer, IL in 2010 and 2011

Main effect	Disease index, DX (CV: 47.80; heritability: 0.200)			
	DF	MS	*F* value	Pr > *F*
Environment (E)	2	27,822.03	292.63	<0.0001
Replication (R)	3	4,082.88	42.94	<0.0001
Genotype (G)	97	312.82	3.29	<0.0001
G × E	194	138.57	1.46	0.0018

**Table 2:** Chromosomal locations and parameters associated with quantitative trait loci (QTL) of disease index (DX) in MD 96-5722 and ‘Spencer’ RIL population evaluated in three different environments in Carbondale, IL in 2010, Valmeyer, IL in 2010 and Valmeyer, IL in 2011

No.	QTL (Env.)	LG/Chr	Peak position (cM)^a^	2-LOD support interval (cM)^b^	Markers interval	Peak LOD^c^	*R*^2^ (%)^d^	Additive effects^e^
1	*qDX001* (*Car.10*)	D1b/Chr_1	15.7	15.60–15.90	ss244562583-ss244554797	7.36	0.92	11.77
2	*qDX002* (*Val.11*)	D1b/Chr_1	15.7	15.40–16.10	ss244562583-ss244554797	3.61	0.50	−0.14
3	*qDX003* (*Car.10*)	N/Chr_3	15.80	15.70–15.90	ss245026358-ss245025977	9.19	0.80	13.24
4	*qDX004* (*Val.11*)	N/Chr_3	16.1	15.90–16.10	ss245025977-ss245026227	3.48	0.10	2.96
5	*qDX005* (*Car.10*)	A1/Chr_5	9.50	9.20–10.00	ss245747167-ss245786667	9.21	0.04	−1.46
6	*qDX005* (*Val.11*)	A1/Chr_5	9.50	8.50–11.70	ss245747167-ss245786667	10.10	0.01	−0.28
7	*qDX006* (*Car.10*)	C2/Chr_6	7.30	7.20–7.30	ss246091245-ss246092064	5.55	0.89	12.71
8	*qDX007* (*Val.10*)	C2/Chr_6	7.30	7.20–7.50	ss246087580-ss246092064	3.38	0.60	12.26
9	*qDX008* (*Val.11*)	C2/Chr_6	7.30	7.20–7.30	ss246091245-ss246092064	7.40	0.54	0.46
10	*qDX009* (*Val.11*)	K/Chr_9	0.50	0.40–0.50	ss246870684-ss246865400	4.13	0.53	0.49
11	*qDX010* (*Car.10*)	O/Chr_10	2.30	1.30–3.30	ss247085505-ss247098566	7.63	0.04	−0.80
12	*qDX011* (*Car.10*)	F/Chr_13	2.70	2.50–2.80	ss247942156-ss247937719	5.04	0.11	−0.08
13	*qDX012* (*Car.10*)	B2/Chr_14	3.00	1.40–4.10	ss248293401-ss248275088	5.96	0.03	0.87
14	*qDX013* (*Car.10*)	B2/Chr_14	15.00	12.90–18.20	ss248293401-ss248275088	17.10	0.03	0.45
15	*qDX014* (*Val.11*)	B2/Chr_14	12.00	10.30–13.00	ss248293401-ss248275088	6.39	6.37	14.33
16	*qDX015* (*Car.10*)	E/Chr_15	2.50	1.50–3.00	ss248604753-ss248616287	4.30	0.05	0.09
17	*qDX016* (*Val.11*)	E/Chr_15	2.50	1.40–2.70	ss248604753-ss248616287	5.89	0.60	3.11
18	*qDX017* (*Car.10*)	J/Chr_16	11.90	11.50–14.00	ss248983974-ss248977568	10.18	0.85	−0.58
19	*qDX018* (*Car.10*)	L/Chr_19	0.10	0.0–0.70	ss250232030-ss250233870	3.12	0.01	0.33

^a^Position of peak LOD value on composite maps described previously (Coles et al. 2010)

^b^The positions that define the two LOD intervals around the position of peak likelihood for the QTL

^c^The log of odds (LOD) value at the position of peak likelihood of the QTL

^d^*R*^2^ estimates the proportion of RIL mean variance (%) explained by the detected QTL

^e^A positive number in additive effect of the QTL indicate that the allele for susceptibility was derived from the line indicated and a negative number mean that the allele for resistance was derived from the line indicated

**Fig. 2:**
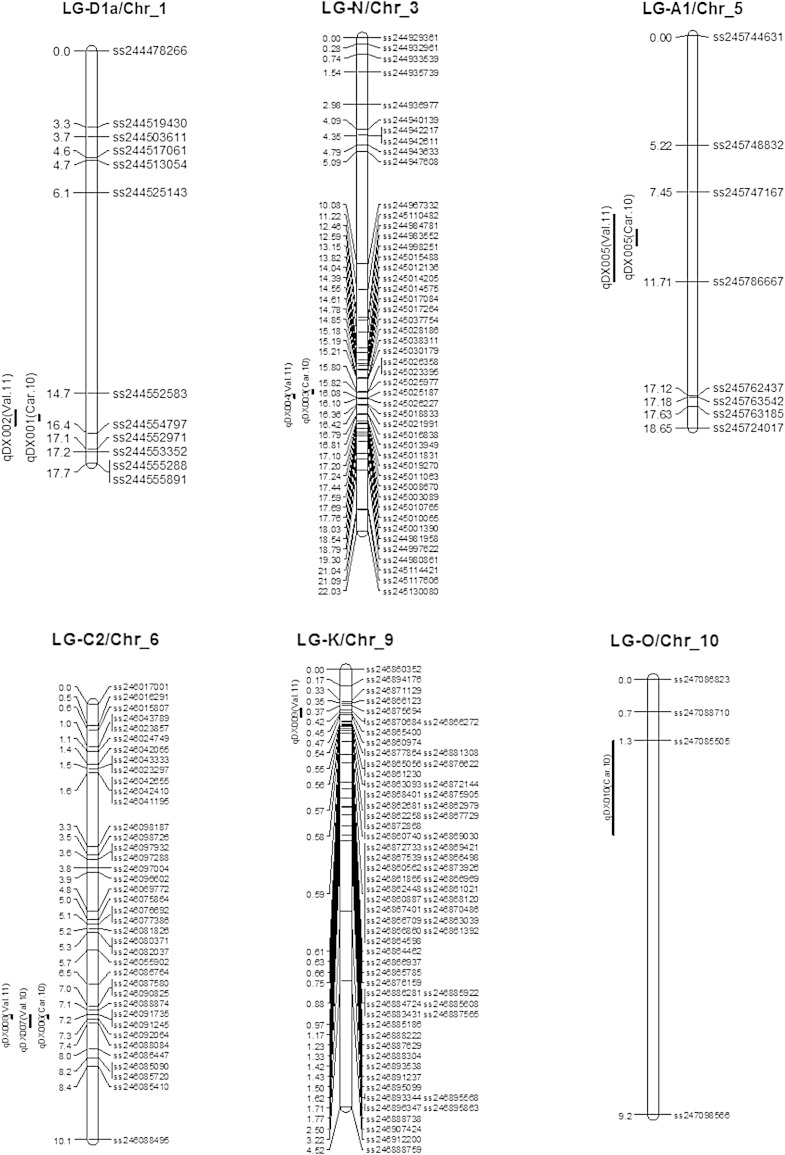

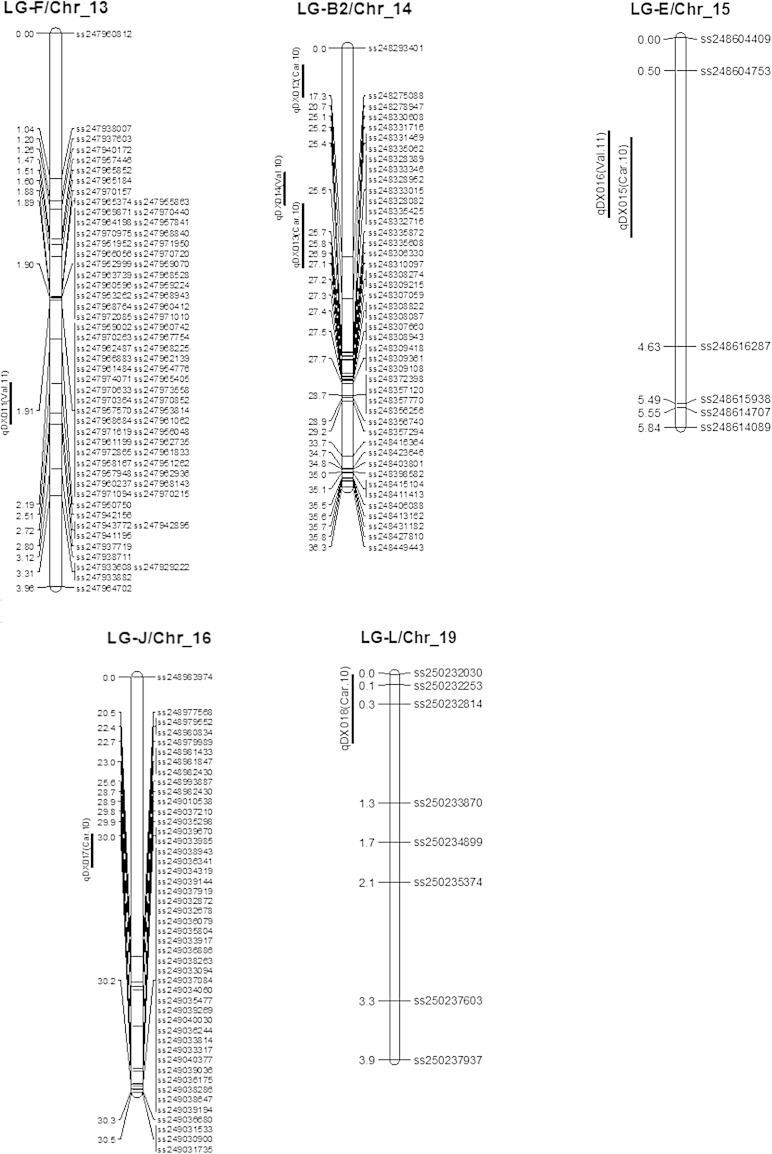
Chromosomal locations of QTL of disease index (DX) in MD 96-5722 and ‘Spencer’ RIL population evaluated in three different environments in Carbondale, IL in 2010, Valmeyer, IL in 2010 and Valmeyer, IL in 2011

Table 2 and Fig. 2 show chromosomal locations and parameters associated with SDS QTL based on DX as calculated from data obtained from three different environments, i.e., Carbondale, IL in 2010 (C-10) and Valmeyer, IL in 2010 (V-10) and Valmeyer, IL in 2011 (V-11). QTL nomenclature was designated based on SoyBase principles and contained abbreviation of disease index (DX) then the serial number of QTL and name of each environment or year in parenthesis. CIM identified 19 QTL for SDS and platted on 11 separate chromosomes of soybean genome (Table 2; Fig. 2). Two QTL for DX [*qDX001* (*Car.10*); *qDX002* (*Val.11*)] were identified on same peak position (15.7 cM) with same marker interval ss244562583-ss244554797 (Table 2; Fig. 2) on LG D1a/Chr_1. QTL for DX had peak LOD score of 7.36 [*qDX001* (*Car.10*)] and 3.61 [*qDX002* (*Val.11*)] with additive effect of 11.77 and −0.14, respectively (Table 2). LG-N/Chr_3 had also two QTL for DX [*qDX003* (*Car.10*); *qDX004* (*Val.11*)]. DX QTL *qDX003* (*Car.10*) and *qDX003* (*Car.10*) had peak LOD score of 9.19 and 3.48 with corresponding additive effect of 13.24 and 2.96 (Table 2). CIM analysis identified two QTL for DX [*qDX005* (*Car.10*); *qDX005* (*Val.11*)] on LG-A1/Chr_5 with the peak LOD score of 9.21 and 10.10 with additive effect of −1.46 and −0.28, respectively (Table 2).

LG-C2/Chr_6 had three QTL for DX [*qDX006* (*Car.10*); *qDX007* (*Val.10*); *qDX008* (*Val.11*)]. The peak LOD scores for DX QTL were 5.55 [*qDX006* (*Car.10*)], 5.60 [*qDX007* (*Val.10*)], and 7.40 [*qDX008* (*Val.11*)] with corresponding additive values 12.71, 0.95 and 0.46. On LG-K/Chr_9, one QTL for DX [*qDX009* (*Val.11*)] was identified with LOD score of 4.13 and additive effect of 0.49. One QTL for DX [*qDX01 0* (*Car.10*)] on LG-O/Chr_10 at two different peak positions (7.3 and 2.3 cM) with marker interval ss247085505-ss247098566 was identified (Table 2; Fig. 2). QTL for DX on LG-O had peak LOD score of 7.63 with additive effect of −0.80 (Table 2). QTL analysis also identified one QTL for DX [*qDX011* (*Car.10*)] on LG-F/chr_13 with peak LOD score and additive of 5.04 and −0.80, correspondingly.

CIM analysis identified three QTL for DX [*qDX012 (Car.10)*; *qDX013* (*Car.10*); *qDX014* (*Val.11*)]. These three QTL had peak LOD scores of 5.96, 17.10 and 6.39 with corresponding additive effect of 0.87, 0.45 and 14.33 (Table 2). LG-E/Chr_15 had two for DX [*qDX015* (*Car.10*); *qDX016* (*Val.11*)] with same marker interval ss248604753-ss248616287. One QTL for DX [*qDX017* (*Car.10*)] was identified on LG-J/Chr_16 having LOD score of 10.18 with additive effect of −0.58. On LG-L/Chr_19, one QTL was identified for DX [*qDX018* (*Car.10*)] having a peak LOD score and additive effect of DX QTL as 3.12 and 0.33, respectively. In total, 19 QTL for DX were identified which explained 47.80 % of total phenotypic variance (Fig. 1) in the *F*_5:7_ RIL population of soybean line MD 96-5722 by Spencer.

## Discussion

MD 96-5722 by ‘Spencer’ RIL population was evaluated for its reaction to SDS in Carbondale and Valmeyer, IL in 2010 and 2011. Across environments some lines in the population had significantly lower DX than MD 96-5722, while no line had a DX higher than ‘Spencer’ (Fig. 1). Genotype, replication and environment (location/year) showed significance at *P* < 0.0001, while genotype × environment (G × E) was significant at *P* ≤ 0.005. Heritability was calculated and displayed low values for DX, which may show that environmental effect has highly influenced the population. Small heritability may also be the consequence of greater additive genetic variation leading to large environmental variances (Barton and Turelli 1989; Roff 1997).

QTL for SDS presented herein are from a high-density SNP-based genetic linkage map of soybeans (Akond et al. 2013) using SoySNP6K Illumina Infinium BeadChip genotyping array, and, in the best of our knowledge, few other scientific groups identified SDS QTL from high-density SNP maps published in soybeans up to date (Choi et al. 2007; Hyten et al. 2010b; Vuong et al. 2010). Among 20 LG of soybean, 11 had SDS QTL of DX at three environments. Many of QTL for DX on each LG over environment were common or on in very adjacent locations. Significant QTL identified here in one environment were confirmed in another, suggesting genetic effects and modes.

Most of SDS QTL identified in our study are on different LG or regions of previously reported ones. Two QTL for DX were identified herein on Chr 1, but on different position than those identified by Kassem et al. (2012) for the same disease in ‘Essex’ by ‘Forrest’ population. There are previous reports of SDS QTL in ‘Essex’ by ‘Forrest’ and ‘Pyramid’ by ‘Douglas’ populations on LG-N (Kassem et al. 2007; Njiti et al. 2002; Hnetkovsky et al. 1996), however, the positions were different probably because those QTL were mapped with SSR markers. Up to date, there are no reports for SDS QTL on LG A1. We also detected three QTL on LG-C2 that were different from SDS QTL on the same linkage group mapped (32.8–39.2 cM positions) by Kassem et al. (2012) in PI 438489B by ‘Hamilton’ RIL population. Iqbal et al. (2001) detected SDS QTL by CIM on LG-C2 on about 13 cM between two microsatellite markers, BARC_Satt277 and BARC_Satt079. SDS (*Rfs*) region on LG-C2 (Kassem et al. 2006; Chang et al. 1996; Hnetkovsky et al. 1996) is very close (4.4 cM) to QTL that is reported herein (2.60–3.40 cM and 7.20–7.30; Table 2; Fig. 2). Kassem et al. (2012) mapped an SDS QTL on LG-O from SNP derived map of PI 438489B by ‘Hamilton’ at 13.5–15.2 cM positions and it is not in the same region of the QTL we identified in this study. We identified several QTL on LG-F and J which were found by Kassem et al. (2007) in ‘Essex’ by ‘Forrest’ population, but in different positions. In this study, new QTL were identified on LG-K (one QTL), B2 (three QTL) and E (two QTL) that have not previously reported. A QTL on LG-L was identified by Kassem et al. (2012), while we are reporting three QTL in different region.

Nineteen SDS QTL identified in this report are on LG or chromosomes that are found in previous studies, but in different positions. Most of previous QTL were mapped through simple linear regression methods (SIM), not by CIM. Different types of markers and different populations that they were grown under greenhouse conditions were used in previous studies. In this study, identification of QTL was performed based SNP genotyping and phenotype data that were collected in two different seasons and locations. Also, SDS rating based on DX as proposed by Njiti et al. (1996) is traditionally used by Southern Illinois University Breeding Program and successfully has led to the development of resistant varieties (Kantartzi et al. 2012a, b). Therefore, QTL identified in this study over three different environments can be useful for soybean breeders developing new SDS resistant cultivars through marker-assisted breeding.
